# *Prunus domestica* Pathogenesis-Related Protein-5 Activates the Defense Response Pathway and Enhances the Resistance to Fungal Infection

**DOI:** 10.1371/journal.pone.0017973

**Published:** 2011-03-23

**Authors:** Ashraf El-kereamy, Islam El-sharkawy, Rengasamy Ramamoorthy, Ali Taheri, Deena Errampalli, Prakash Kumar, Subramanian Jayasankar

**Affiliations:** 1 Department of Plant Agriculture, University of Guelph, Vineland, Ontario, Canada; 2 Department of Biological Science, National University of Singapore, Singapore, Singapore; 3 Department of Plant Agriculture, University of Guelph, Guelph, Ontario, Canada; 4 Southern Crop Protection and Food Research Centre, Agriculture and Agri-Food Canada, Vineland Station, Ontario, Canada; University Medical Center Groningen, Netherlands

## Abstract

Pathogenesis-related protein-5 (PR-5) has been implicated in plant disease resistance and its antifungal activity has been demonstrated in some fruit species. However, their roles, especially their interactions with the other defense responses in plant cells, are still not fully understood. In this study, we have cloned and characterized a new PR-5 cDNA named *PdPR5-1* from the European plum (*Prunus domestica*). Expression of *PdPR5-1* was studied in different cultivars varying in resistance to the brown rot disease caused by the necrotrophic fungus *Monilinia fructicola.* In addition transgenic *Arabidopsis,* ectopically expressing *PdPR5-1* was used to study its role in other plant defense responses after fungal infection. We show that the resistant cultivars exhibited much higher levels of transcripts than the susceptible cultivars during fruit ripening. However, significant rise in the transcript levels after infection with *M. fructicola* was observed in the susceptible cultivars too. Transgenic *Arabidopsis* plants exhibited more resistance to *Alternaria brassicicola*. Further, there was a significant increase in the transcripts of genes involved in the phenylpropanoid biosynthesis pathway such as *phenylalanine ammonia-lyase* (*PAL*) and phytoalexin (camalexin) pathway leading to an increase in camalexin content after fungal infection. Our results show that *PdPR5-1* gene, in addition to its anti-fungal properties, has a possible role in activating other defense pathways, including phytoalexin production.

## Introduction

European plum (*Prunus domestica*) is a commercially important stone fruit species. Similar to other fruit crops, these plums are also affected by several pathogenic microbes during ripening, causing significant economic losses all around the world. One of the major diseases, brown rot, is caused by a fungus *Monilinia fructicola* which attacks the fruits at the onset of ripening and continues to be a menace through post harvest storage and shipping. In addition, this fungus also causes severe blossom blight from the mummified fruits that carry the fungal spores thorough winter. Conventional breeding for disease resistance is a lengthy as well as a low probability process in such tree fruits and thus chemical control is the only alternative available for the growers. With increasing public pressure to reduce chemical sprays in such fresh products, understanding the resistance mechanisms at molecular level, which can then be functionally translated to successful cultivars, is an important area of research. It has been demonstrated in economically important species such as grapes that fruit ripening is accompanied by increased expression of potentially fungal disease resistance genes such as chitinases, glucanases and thaumatin-like proteins [1], [2] all of which belong to the family of pathogenesis-related (PR) proteins.

PR proteins comprise of seventeen groups, which are mostly induced by pathogen infection and elicitor treatment; and they contribute to disease resistance in many plant species [3]–[5]. Several PR proteins belonging to the group 5 (PR5) exhibit amino acid and structural similarity to an intensely sweet tasting protein, thaumatin, from the fruits of the West African shrub, *Thaumatococcus daniellii*, and hence are called thaumatin-like proteins (TLPs) [6]. However, all of the TLPs lack the sweet tasting property of thaumatin [7]. PR5 family includes basic and acidic members according to their isoelectric points, although they show similar activity. Some of the recombinant PR5 proteins that have been purified are antifungal [8]–[15]. PR5 proteins generally exert their antifungal activity through a very fast and dramatic increase in the permeability of the pathogen's plasma membrane, by disrupting the lipid bi-layer and creating trans-membrane pores. Hence, some of them are also called permatin [8]. Osmotin, another PR5 protein, interferes with pathogen signal transduction pathway to enhance fungal cell susceptibility [16]. PR5 proteins in fruit species like cherries, tomatoes, grapes and banana are often induced upon fruit ripening, when the sugar increases, suggesting a possible secondary role in ripening in addition to its antifungal activity [1], [2], [17], [18].

Over-expression of PR5 proteins in transgenic plants have generally resulted in enhancing disease resistance in some plant species. For example, potato osmotin enhances resistance to potato late blight pathogen *Phytophtohra infestans* [19]; the rice *TLP-D34* enhances resistance to the sheath blight pathogen, *Rhizoctonia solani* [20]; rice *TLP* enhanced resistance to the head blight pathogen *Fusarium graminearum* [21]–[23]. Constitutive expression of *Vitis vinifera* TLP, VVTL-1 plays an important role in grape resistance to anthracnose [14] and transgenic grapes expressing VVTL-1 exhibit sustained resistance to several fungal pathogens such as *Uncinula necator* and *Botrytis cinerea* [24].

Upon pathogen infection, plants initiate a large spectrum of defense responses involving several pathways including the deployment of PR proteins. Other defense mechanisms exerted by plants include the deposition of mechanical barriers like phenylpropanoids, carbohydrates and hydroxyproline-rich glycoproteins within cell walls to limit invasion by fungal hyphae [25]. Elicitor and fungal infection stimulates the transcription of several genes in the phenylpropanoid biosynthesis pathway such as *phenylalanine ammonia-lyase* (*PAL*), *chalcone synthase* (*CHS*), *cinnamic acid 4-hydroxylase* (*CA4H*), and *isoflavone reductase* (*IFR*), which code for key enzymes involved in the synthesis of lignin precursors and the synthesis of flavonoids and isoflavonoid-derived phytoalexins [26]–[28]. Further, plants also synthesize small secondary metabolites such as phytoalexins that are fungitoxic [29] and are associated with disease resistance [30]–[32]. The phytoalexin, camalexin [3-(2-thiazolyl) indole] was first isolated from the leaves of *Camelina sativa* and elicited by the fungus *Alternaria brassica* [33]. It is the major *Arabidopsis thaliana* phytoalexin that exhibits antifungal activity similar to the systemic fungicide thiabedazole [34], [35]. Several lines of evidence revealed the importance of the camalexin in disease resistance [36], [37]. In addition, *Arabidopsis* mutants defective in the camalexin pathway such as *pad1, pad2* and *pad3* show susceptibility to the different pathogens [38], [39]. Although several PR5 proteins have been cloned and characterized from different plant species, their physiological role in other defense responses (e.g. phytoalexin) in plant cells is not fully understood. In the present study, we have cloned and characterized a gene for European plum PR5 protein and made an attempt to study its role in the camalexin pathway.

## Results

### In-silico analysis of PdPR5-1

**Figure 1:**
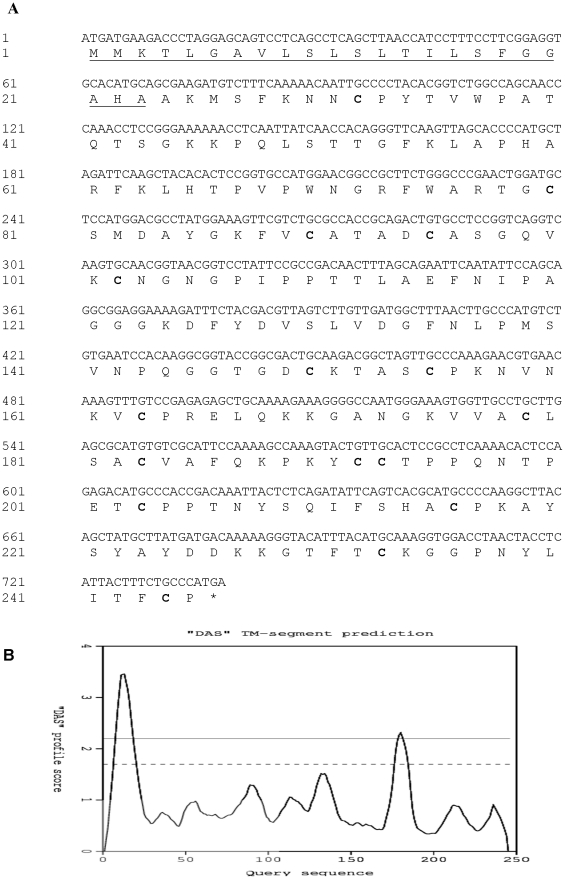
Sequence and structure of *PdPR5-1*. (**A**) Nucleic and deduced amino acid sequences of *PdPR5-1*. Deduced amino acids for the protein are shown under the nucleic acid sequence. The predicted signal peptide is underlined. The sixteen conserved cysteine residues are in bold. (**B**) The transmembrane domains in the PdPR5-1 as predicted by DAS-Trans-membrane prediction server.

**Figure 2:**
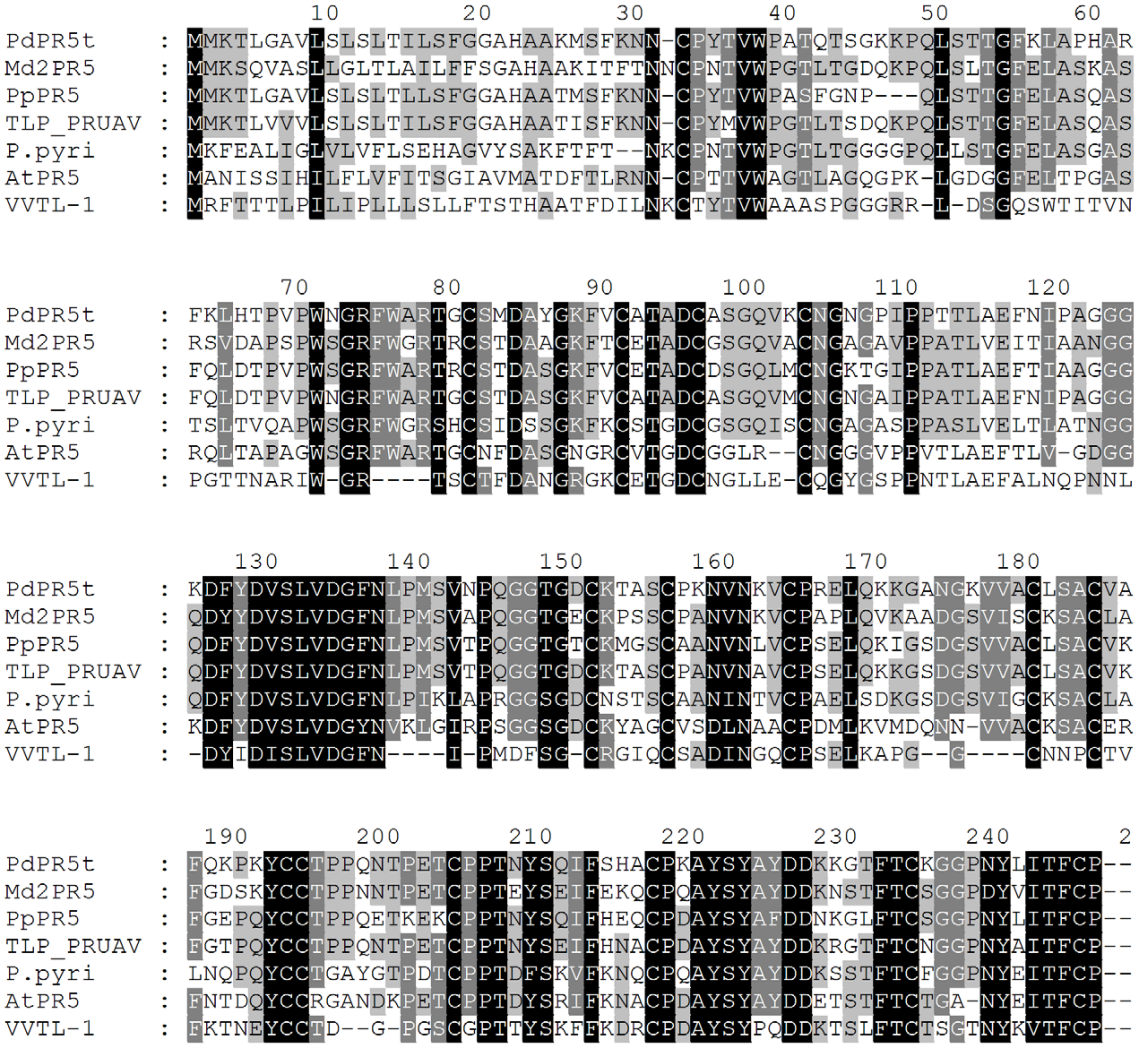
Multiple amino acid sequence alignments of *Prunus domestica* PR5; PdPR5-1 (HM853674) with the closely related sequences, *Malus domestica* Md2PR5 (AJ243427), *Prunus persica* PpPR5 (AAM00215.1), *Prunus avium* TLP_PRUAV (P50694.1), *Prunus pyrifolia* P.pyri (BAA28872.1), *Arabidopsis thaliana* AtPR5 (At1g75040) and *Vitis vinifera* VVTL-1 (AAB61590). Conserved residues are shaded in black. Dark grey shading indicates similar residues in six out of seven of the sequences, and light grey shading indicates similar residues in five out of seven of the sequences.

Sequence analysis of the full length *PdPR5-1* cDNA revealed that it comprises of 735 bp open reading frame (245 amino acids). It also showed the presence of 16 cysteine, typically conserved in all PR5 proteins (Figure 1A) as previously reported [10], [18]. The derived amino acid sequence indicates the presence of potential trans-membrane segments between 7^th^ and 20^th^ (AVLSLSLTILSFGG) and between 177^th^ and 184^th^ (VACLSACV) amino acids (Figure 1B) as predicted by DAS-trans-membrane prediction server (http://www.sbc.su.se/~miklos/DAS/). The predicted molecular weight of the complete protein is 26.136 kDa with an isoelectric point (IEP) of 9. However, the amino acid sequence indicates the presence of a signal peptide sequence followed by a cleaving site between the 23^rd^ and 24^th^ amino acids (Figure 1A). Thus the mature protein should be composed of 222 amino acids, with an apparent molecular weight of 23.8 kDa and a pI of 8.89, suggesting this to be a basic protein. The PdPR5-1 shares high sequence similarity with previously reported PR5 proteins from other species such as *Malus domestica* (67%), *P. persica* (77%), *P. avium* (83%), *Vitis vinifera* (64%), *Pyrus pyrifolia* (56%) and *Arabidopsis thaliana* (51%) (Figure 2). Although PR5 was thought to be a single member gene family earlier [2], blast search of the *Arabidopsis* genome revealed the presence of six homologues to the PdPR5-1 with the following accession numbers: At1g75040, At1g20030, At1g75050, At1g75030, At1g75800 and At4g38660, which have sequence similarity ranging from 47–52% to the PdPR5-1.

### Expression of *PdPR5-1* after fungal infection in plums

**Figure 3:**
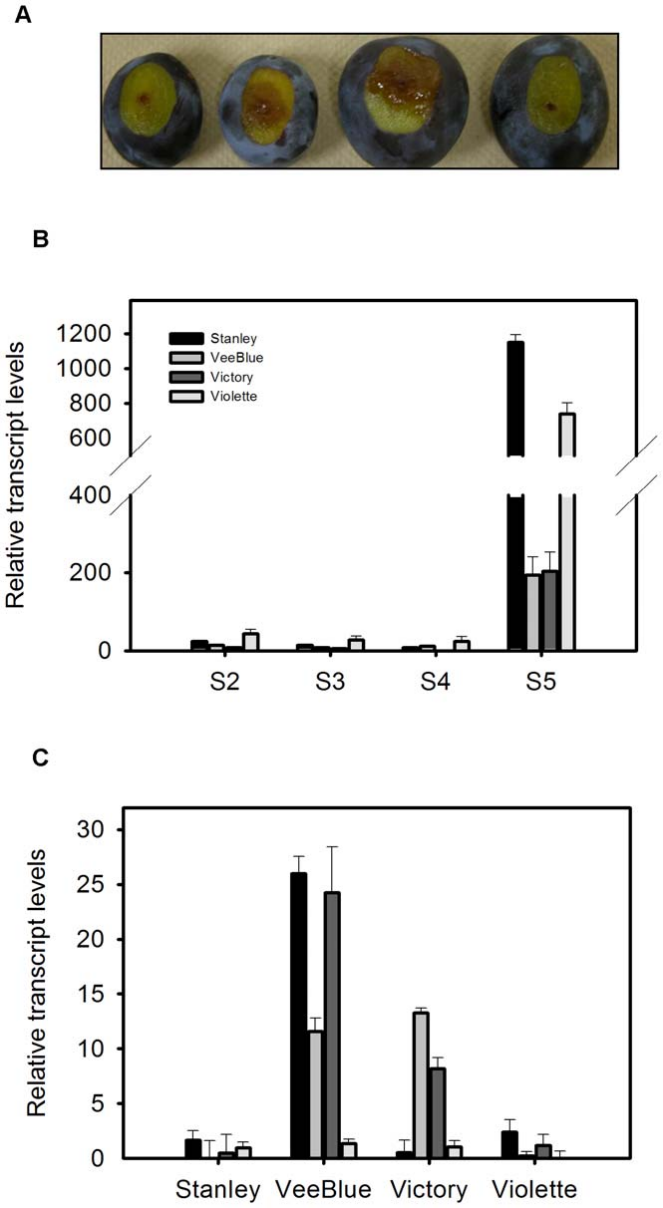
Transcript levels of *PdPR5-1* in different plum cultivars during ripening and after fungal infection. (**A**) Lesion development in four European plum varities three days after the infection. (**B**) Accumulation of the transcript level of *PdPR5-1* during fruit maturation in four European plum varities. S2 = Before pit hardening; S3 = After pit hardening; S4 = Fully mature stage and S5 = Ripe stage (**C**) Induction of the *PdPR5-1* in the susceptible and resistant plum varieties from one to four days post-inoculation with *Monilinia fructicola.*

To study the role of the PR5 protein in plums, we analyzed its transcript accumulation in four European plum cultivars with varying degrees of resistance to brown rot disease. Stanley and Violette are less susceptible varieties than Veeblue and Victory. It is clear that the lesion diameter is bigger in the susceptible varieties three days after inoculation with *M. fructicola* (Figure 3A). We monitored the transcript levels of *PdPR5-1* during fruit maturation and ripening as well as after *M. fructicola* infection. Quantitative Real Time PCR analysis revealed that *PdPR5-1* transcript level is generally low at the early, after pit hardening (S2 & S3), and mature green stages (S4) and dramatically rises during fruit ripening (S4) in all four varieties. Interestingly, we found that at fruit harvest stage *PdPR5-1* expression is higher in the resistant varieties, Stanley and Violette, compared to susceptible varieties Veeblue and Victory (Figure 3B), indicating that *PdPR5-1* might be developmentally regulated and has a role during fruit ripening. However, after fungal infection, the expression pattern of *PdPR5-1* was different and contradicting the constitutive levels. In the susceptible cultivars, Veeblue and Victory, the transcript levels rapidly increased during the first three days after infection, while the resistant varieties, Stanley and Violette showed only a marginal increase (Figure 3C). The induction of *PdPR5-1* after fungal infection also correlates well with lesion diameter observed in the four varieties, three days after the infection (Figure 3A).

### Enhancing disease resistance by *PdPR5-1* in transgenic *A. thaliana*

In order to evaluate the biological function of *PdPR5-1* in plants, we transformed *Arabidopsis* (ecotype Colombia) with this gene. We used *Arabidopsis* for the functional analysis as the transformation efficiency is low in European plums and also keeping in mind the long pre-bearing age of this crop. Putative *Arabidopsis* transgenic plants were transferred to growth chambers and kept at 16 h/8h photoperiod.

**Figure 4:**
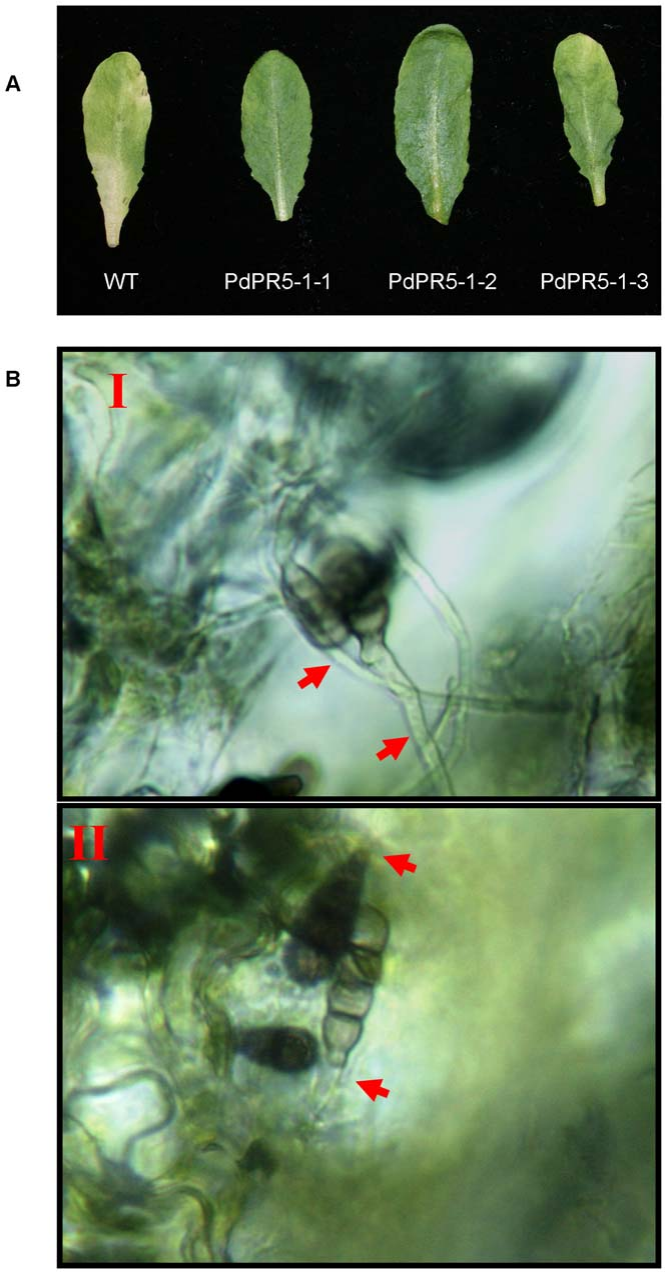
Overexpression of *PdPR5-1* in *Arabidopsis* increases the resistance to *Alternaria brassicicola* infection. (A) In vitro inoculation of *A. brassicicola* on *Arabidopsis* leaves. Leaves were infected with *A. brassicicola* spores and incubated in humid conditions for symptom development. The wild type (WT) leaves exhibited clear disease symptoms [yellowing on the margins and spots in the lamina] within 3 days of inoculation, while the *PdPR5-1* transgenics significantly delayed the symptom development. (B) When examined using a microscope, uninhibited germination and growth of *A. brassicicola* spores can be seen on WT leaves (I) while the spores on *PdPR5-1-1* leaves showed poor or no germination [II, arrow] further confirming that PdPR5-1 increases resistance to *A. brassicicola* in transgenic *Arabidopsis*.

To see if *PdPR5-1* plays any role in fungal disease resistance in transgenic *Arabidopsis*, these plants were subjected to *in vitro* leaf infection assay. Fully developed rosette leaves from all three transgenic lines and Columbia plants were collected and inoculated with a virulent strain of *Alternaria brassicicola*. Three days after infection, disease symptoms (visible as necrotic lesions and yellowing) were observed on the leaves of the WT, whereas the leaves of the three transgenic lines did not show such symptoms (Figure 4A). On close examination, fine spots of fungal infection could be seen, but the disease did not set in as in the WT. Enhanced fungal disease resistance in transgenic crop plants expressing their respective PR5 genes has been shown in potato [19], rice [20], wheat [21], [22] and bent grass [40]. It has been demonstrated that PR5 proteins inhibit the hyphal growth of fungal spores at the points of infection. To verify this, we looked at the hyphal growth in the infected regions of the WT and transgenic leaves, three days after the infection. As expected, the *Alternaria* hyphae were spreading in an uninhibited manner over the infected area of WT leaves, whereas it was significantly inhibited in the leaves of the transgenic plants (Figure 4B).

### *PdPR5-1* and *Arabidopsis* defense responses during fungal infection

**Figure 5:**
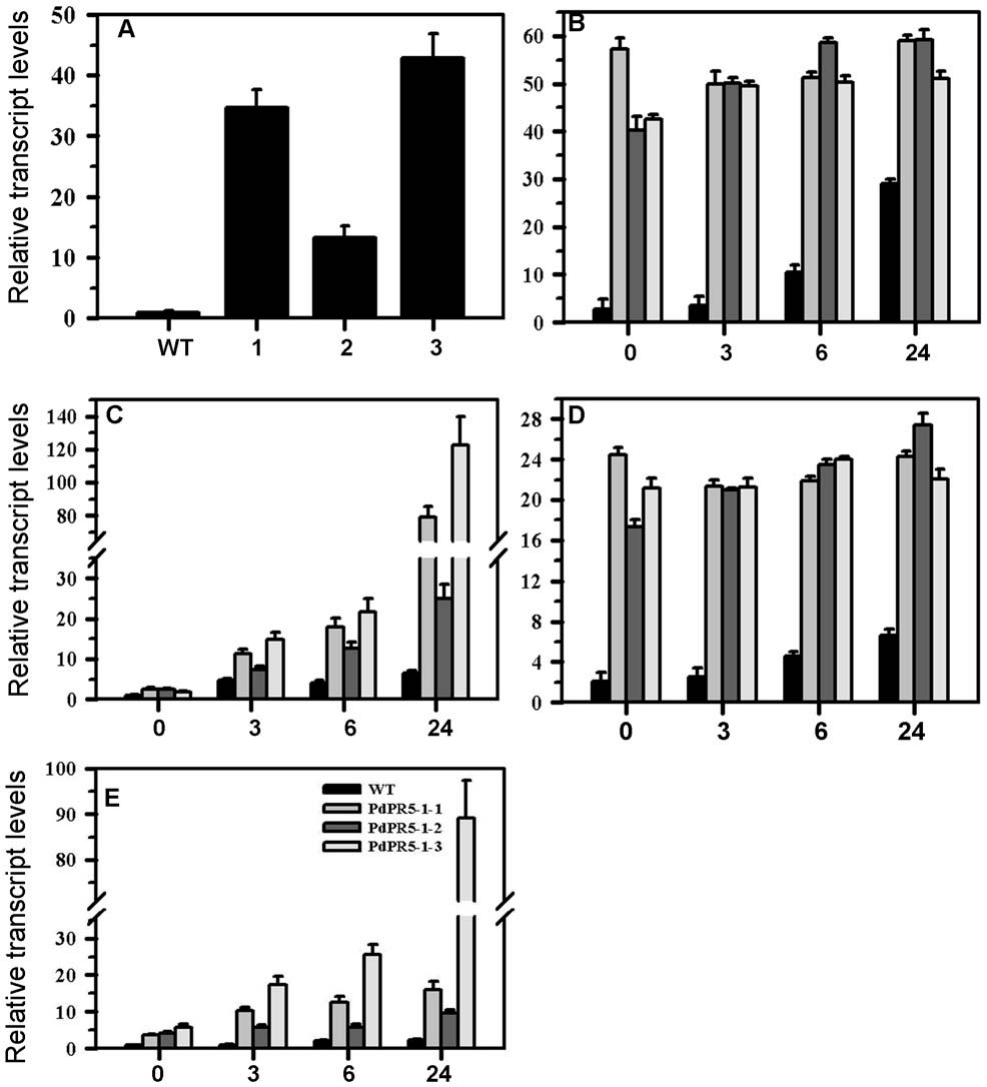
Relative transcript levels of *PdPR5-1* and camalexin intermediaries in *Arabidopsis.* (**A**) Expression of *PdPR5-1* in the three transgenic *Arabidopsis* lines; WT = Wild type, 1 = PdPR5-1-1, 2 = PdPR5-1-2, 3 = PdPR5-1-3; (**B**) Expression of *PAL*, (**C**) *CYP79B2*, (**D**) *CYP79B3* and (**E**) *PAD3* in *Arabidopsis* WT and transgenic lines. X-axis indicates time in hours after inoculation with *Alternaria brassicicola.*

The presence of *PdPR5-1* in *Arabidopsis* transgenic plants was confirmed by PCR analysis and the expression of *PdPR5-1* in the leaves was monitored using qRT-PCR. Three independent transformants were selected and used for further analysis presented in this study (Figure 5A).

In addition to the induction of pathogenesis related proteins upon fungal infection, the host cells also activate several other lines of defense enhancing the resistance. Despite the well known role of PR5 as antifungal protein, little is known about its role in other defense pathways. It has been shown that treatment of plants with elicitors of defense responses can result in the accumulation of PR proteins and antimicrobial compounds such as phytoalexins [41]. One of the common defense mechanisms activated following elicitor treatment and fungal infection in plants is the phenylpropanoid pathway [27]. Hence, we decided to examine if there is any change in the phenylpropanoid pathway in the transgenic plants after fungal infection and compared it with WT. As a first step, we examined the transcription level of *Phenylalanine ammonia lyase* (*PAL*), a key gene in the phenylpropanoid pathway in the WT and transgenic plants after fungal infection (Figure 5B). All the transgenic lines showed a higher level of *PAL* (40- to 60-fold) from 3 to 24 hours post inoculation. However, a significant induction of *PAL* transcript levels was observed in the WT plants only 24 hours after inoculation. These results further supported our hypothesis that PR5 activates other defense mechanisms in plants.

Recently, it has been suggested that genotypic resistance in tobacco is correlated with the accumulation of scopoletin (a phytoalexin) and PR proteins [36], indicating that PR proteins might interact with phytoalexins. Camalexin is the main phytoalexin involved in *Arabidopsis* defense, as it is fungi-toxic and thus associated with disease resistance [29]–[32]. Several intermediate signals leading to the biosynthesis of camalexin in *Arabidopsis* have been identified. Among the intermediaries, three cytochrome P450 enzymes CYP79B2, CYP79B3 and CYP71B15 (PAD3) play a major role in camalexin biosynthesis in *Arabidopsis* [42], [43]. Mutants defective in any of these three genes in the camalexin pathway are defective in camalexin synthesis and susceptible to fungal infection. Hence, we attempted to follow these three genes leading to camalexin biosynthesis in the WT and *PdPR5-1* transgenic plants. Our analysis revealed that the CYP79B2 which is the first enzyme in this pathway is significantly higher in transgenic lines after *Alternaria* infection. However, even after 24 hours only a slight increase in CYP79B2 transcripts was seen in WT (Figure 5C). CYP79B3 transcripts were constitutively high in the transgenic lines and increased only to a limited scale in WT even after 24 hours post infection. The general trend didn't indicate that this gene is induced by pathogen infection although one line (PR5-1-2) showed a linear response with time (Figure 5D). The expression of the last gene in camalexin pathway, *PAD3*, was also higher in the transgenic lines than the WT before infection and continued to increase steadily after infection only in the transgenic lines. The increase in *PAD3* transcripts paralleled that of *CYP79B2* in all three transgenic lines and interestingly, the transcripts of *PdPR5-1* also exhibited the same pattern (Figure 5 A, C and E). Collectively, these data suggest that *PdPR5-1* intervenes in the existing camalexin mechanism and affirms the view that CYP79B2 and PAD3 are the critical intermediaries in the camalexin pathway. However, gene expression may not mean much in disease resistance if they are not translated into the final product. Hence, we quantified the camalexin content in *Arabidopsis* in response to pathogen infection.

### Camalexin estimation in *PdPR5-1* transgenic *Arabidopsis* lines

**Figure 6:**
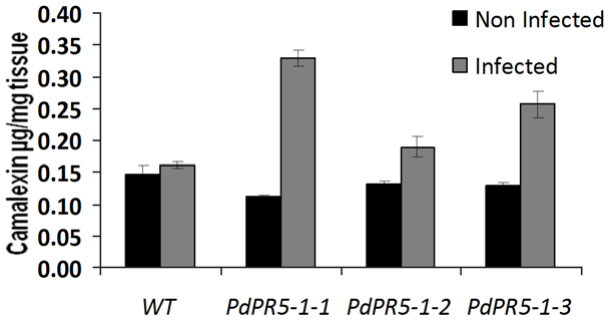
Camalexin content in the leaves of transgenic *Arabidopsis* plants expressing the plum *PdPR5-1* gene. Leaves were collected three days after inoculation with *Alternaria brassicicola* from the wild type and three independent transgenic lines.

Basal camalexin levels were slightly lower in all the three transgenic lines than the WT. This was a bit surprising, considering that all the transgenic lines had significantly higher levels of transcripts for the intermediates including the key PAD3 enzyme. Infection with *A. brassicicola* changed the dynamics of camalexin production dramatically. While the increase in the WT was not pronounced, all the three transgenic lines showed a marked increase in camalexin, with PR5-1-3 exhibiting a threefold increase (Figure 6). It is also interesting to note that this increase in camalexin negatively correlates with the expression of disease symptoms (Figure 4A).

### *PdPR5-1* promoter analysis

A 912 bp of the *PdPR5-1* promoter region was cloned as described in the material and methods section. To understand the regulation of the *PdPR5-1* gene, an in silico analysis of the promoter (912 bp) was carried out using PLACE (http://www.dna.affrc.go.jp/PLACE/)-and PLANT-CARE (http://bioinformatics.psb.ugent.be/webtools/plantcare/html/) web sites (Table 1 & 2). The analysis of the *PdPR5-1* promoter revealed the presence of several *cis*-acting regulatory elements (CAREs) and transcription factor binding sites (TFBS) related to different physiological responses as shown in Tables 1 & 2. For example, this promoter contains CAREs related to all important plant hormones such as abscisic acid (ABA), gibberellic acid (GA), auxin, cytokinin, salicylic acid (SA) and jasmonic acid (JA). Additionally, the *PdPR5-1* promoter has TFBS for many transcription factors already identified in other organisms and have a role in the activation of the promoter of some genes involved in biotic and abiotic stress responses, disease resistance or hormonal response. Those transcription factors include DOF, MYB, AP2 and WRKY.

**Table 1:** *PdPR5-1* promoter analysis using PLACE website.

Site Name	Position	Strand	Sequence	Function
BIHD1-OS	-187, -463	+	TGTCA	BELL homeodomain transcription factor involved in disease resistance responses
Box Lcore DCPAL	-264	−	ACCWWCC	MYB; R2R3 type; PAL: Elicitor; UV-B; Dilution;
CCA1ATLHCB1	-141	+	AAMAATCT	myb-related transcription factor
CPBCSPOR	-638	−	TATTAG	Critical for Cytokinin-enhanced Protein Binding in vitro
DOF-CoreZM	-831, -440, -267, -85, -77, -46, -896, -419, -368, - 364	+	AAAG	Dof proteins binding site
DPBF-CoreDCDC3	-621, -121	+	ACACNNG	bZIP transcription factors, DPBF-1 and 2
EECCRCAH1	-432, -226	+	GANTTNC	Binding site of Myb transcription factor LCR1
MYB-1AT	-725	−	WAACCA	MYB recognition site found in the promoters of the dehydration-responsive gene rd22
MYB2-CONSENSUSAT	-112	−	YAACKG	MYB recognition site found in the promoters of the dehydration-responsive gene rd22
MYB-CORE	-112	+	CNGTTR	water stress in Arabidopsis A petunia MYB protein (MYB.Ph3)
MYB-PZM	-264	−	CCWACC	Core of consensus maize P (myb homolog) binding site;
MYC-ATERD1	-842	−	CATGTG	MYC recognition sequence necessary for expression of erd1
MYC-AtRD22	-842	+	CACATG	Binding site for MYC (rd22BP1) in Arabidopsis dehydration-resposive gene
MYC-CONSENSUSAT	-842, -609, -553, -213, -112, -842, -609, -553, -213, -112	+	CANNTG	MYC recognition site
PYRIMIDINE Box OS RAMY1A	-832, -268	−	CCTTTT	Gibberellin-respons cis-element of GARE
RAV1-AAT	-780, -762, -572, -319, -216	+	CAACA	RAV1 and AP2-like proteins binding sequence
T/GBOXATPIN2	-389	+	AACGTG	"T/G-box" Involved in jasmonate (JA)
TAAAG-STKST1	-47	+	TAAAG	StDof1 protein site controlling guard cell-specific gene expression
TCA1-MOTIF	-547	−	TCATCTTCTT	salicylic acid-inducible expression of many genes
WBox-ATNPR1	-464	+	TTGAC	salicylic acid (SA)-induced WRKY DNA binding
Wbox-HVISO1	-559, -845, -738, -560	+	TGACT	WRKY binding site involved in sugar signaling in barley
Wbox-NTERF3	-559, -845, -738	+	TGACY	W box
WRKY71-OS	-559, -463, -844, -737, -186	+	TGAC	TGAC-containing W box elements

**Table 2:** *PdPR5-1* promoter analysis using PLANT CARE website.

Site Name	Position	Strand	Sequence	Function
ABRE	-255	+	CCTACGTGGC	cis-acting element involved in the abscisic acid responsiveness
BRE	-726	+	TGGTTT	cis-acting regulatory element essential for the anaerobic induction
AT-rich element	-353	−	ATAGAAATCAA	binding site of AT-rich DNA binding protein (ATBP-1)
ATGCAAAT motif	-874	+	ATACAAAT	cis-acting regulatory element associated to the TGAGTCA motif
Box 4	-885	+	ATTAAT	part of a conserved DNA module involved in light responsiveness
G-Box	-390	−	CACGTT	cis-acting regulatory element involved in light responsiveness
GAG-motif	-705	−	AGAGATG	part of a light responsive element
GATA-motif	-440	+	AAGATAAGATT	part of a light responsive element
GT1-motif	-208	−	AATCCACA	light responsive element
HSE	-165	−	AGAAAATTCG	cis-acting element involved in heat stress responsiveness
I-box	-175	−	GTATAAGGCC	part of a light responsive element
MBS	-113	−	CAACTG	MYB binding site involved in drought-inducibility
MNF1	-96	+	GTGCCC(A/T)(A/T)	light responsive element
MRE	-642; -362	+	AACCTAA	MYB binding site involved in light responsiveness
Skn-1_motif	-738	+	GTCAT	cis-acting regulatory element required for endosperm expression
TC-rich repeats	-550; -303	−	ATTTTCTTCA	cis-acting element involved in defense and stress responsiveness
TGA-element	-578	+	AACGAC	auxin-responsive element

## Discussion

In this study we cloned a full length cDNA of a new *PR5* gene from the European plum, and designated it as *PdPR5-1*. Structural analysis confirms it to be a thaumatin-like protein (TLP). It is well known that PR5 proteins are defense proteins, have membrane binding domains which affect the permeability, thus impeding the growth of invading microbes (Grenier *et al*., 1999). In some fruit species, TLPs are induced with the onset of ripening [2], [17], [18] and thus it was thought to be a developmentally controlled gene with fruit-specific expression [2]. In the present study, since there is a pronounced increase in the *PdPR5-1* transcript level with the onset of fruit ripening in all studied cultivars, it is likely that this isoform is ripening related. Constitutively high levels of *PdPR5-1* transcripts were seen in the two resistant genotypes, Stanley and Violette when compared to the susceptible genotypes Veeblue and Victory. In addition, after infection with *M. fructicola*, the transcript levels dropped immediately in the resistant genotypes but started to increase in the susceptible genotypes, indicating a potential early role for this gene in brown rot resistance in plums. Earlier works suggest that TLPs are induced after fungal infection [8], [11], [13]–[15], but genotypic variability (especially in fruit species) and their downstream action is largely unknown.

Several PR5 proteins identified so far have been implicated in plant defense, only a few functional studies have been conducted demonstrating the PR5 proteins to be anti-fungal, either using recombinant proteins [14] or through transgenic approaches [20], [22]. Thus it is imperative to demonstrate the function of any new member of this group. Using an in vitro detached leaf assay we showed that the transgenic *Arabidopsis* plants harboring *PdPR5-1* exhibit increased resistance to infection by *Alternaria brassicicola* (Figure 4), thus proving the functionality of this gene.

In most situations pathogen attack is not a onetime event. It will be a sustained action with spores constantly trying to gain entry into the host. This is especially true in fruits, as latent infections are very common with diseases such as brown rot. As our data suggests, *PdPR5-1* (or TLPs in general) constitutes only a first line of defense. In the resistant genotypes this first line of defense could successfully ward of the microbial threat, but in doing so drains the fruit of the *PdPR5-1* transcripts within 24 hours (Figure 3A, B). Along with the PR proteins, the phenylpropanoid and the phytoalexin biosynthesis are common defense pathways that are induced upon fungal infection in most plant species [36], [44], [45]. It has also been demonstrated that several elicitors that induce PR proteins have also concomitantly induced phytoalexins in some species [36], [41] thus increasing the resistance to pathogen. But to our knowledge the connection between the induction of PR proteins and other defense response pathways including phytoalexins has not been understood. This encouraged us to explore if *PdPR5-1* also affects the phytoalexin biosynthesis.

Camalexin, the main phytoalexin in *Arabidopsis*, is readily induced by fungal and bacterial pathogens [46]. Biosynthesis of camalexin involves some cytochrome P450 (CYP) genes. One of the proposed models for camalexin biosynthesis involves CYP79B2 and CYP79B3 to convert tryptophan into dihydro camalexic acid, which is then converted by CYP71B15 (PAD3) to camalexin [47]. We examined the expression levels of these genes, because they have a role in antifungal defense [48]. Our results showed that transcripts of all the three enzymes significantly increased in the *PdPR5-1* transgenic *Arabidopsis* lines, even before fungal infection and *CYP79B2* and *PAD3* transcript levels increased after fungal infection in a steady manner (Figure 4). Thus, it appears that *PdPR5-1* affects this pathway of camalexin production in *Arabidopsis*. It also suggests that increased expression of CYP79B2 results in a concomitant increase in PAD3 and enhanced camalexin biosynthesis. These results are in agreement with an earlier study [49], where they have shown significant increase in *CYP79B2* (and not *CYP79B3*) transcripts after elicitor treatment. However, other studies have indicated that changes in *CYP79B2* and/or *CYP79B3* need not necessarily alter *PAD3* expression [50] and activating the expression of *PAD3* is not a fool proof evidence to activate camalexin biosynthesis in some cases such as SA treatment, since camalexin synthesis could be regulated by another unknown mechanism [51]. Collectively, all these studies including ours point to the fact that these intermediaries are definite, but not exclusive players in camalexin biosynthetic pathway. It would have been desirable if the camalexin induction in the *Arabidopsis* PR5 knockout mutant was analyzed. However, the presence of more than six *PR5* orthologs in *Arabidopsis* make the possibility of redundancy higher and correlation of camalexin synthesis may not necessarily be easily interpreted.

Based on the higher expression levels of camalexin biosynthetic enzymes, especially *PAD3*, we anticipated that the basal camalexin levels will be higher in the *PdPR5-1* transgenics. To our surprise it was lower in all three lines before fungal infection. However, following *Alternaria* infection camalexin levels increased significantly only in transgenic lines, suggesting an indirect role for *PdPR5-1* in camalexin synthesis (Figure 6). It has been observed that certain mutants such as salicylic acid induction-deficient *(sid)* and *ups-1* mutants exhibit normal levels of PR5 and concomitant levels of camalexin after pathogen infection [31], [52]. In addition, factors such as oxidative stress that induce PR5 levels also tend to increase camalexin levels [53]. Natural variation for camalexin biosynthesis exists among different ecotypes and in response to different treatments [54]. Further, they also indicate that such natural variation is more likely due to elicitor recognition and signal transduction than differing biosynthetic efficiencies. It is likely that the *PdPR5-1* transgenics recognize pathogen infection quickly and induce camalexin production as a second wave of defense. Camalexin is usually produced around the infection sites [54] and fungal products can degrade camalexin quickly, thus helping in the invasion. In the WT since the infection is rapid and sustained than the transgenics, there is a likelihood of camalexin degradation. Thus, camalexin increase in the *PdPR5-1* transgenics after fungal infection may be attributed to an improved signal transduction, although this needs further investigation. Recent studies have also confirmed that the expression of camalexin biosynthetic genes need not necessarily correlate with the actual metabolite and that there may be post-transcriptional changes or there could be yet unknown players in its production [37]. Although the present study focused on the camalexin pathway, we do not exclude the existence of other mechanisms by which *PdPR5-1* enhances the plant resistance to fungal infection.

To our knowledge, *PR5* promoter has not been analyzed in detail in any of the fruit crops. This encouraged us to do a preliminary analysis of the *PdPR5-1* promoter. As expected, we discovered the presence of several *cis*-regulatory elements with potential involvement in biotic and abiotic stress responses. Notable among these are the *cis*-elements involved in defense responses such as salicylic acid, T/G box involved in jasmonate response, binding sites for MYB, MYC, WRKY and AP2 like proteins. The presence of such *cis*-elements indicates the potential for *PdPR5-1* to interact with other regulatory pathways.

### Conclusions

Our results suggest that besides the well known antifungal activity for the PR5 proteins, they might have additional functions in plant cells. Overexpression of PdPR5-1 protein favorably alters the regulation of phytoalexin accumulation, thus constituting an active second line of defense. In addition, PR5 overexpression in other species has increased the H+−ATPase activity, improved seed germination and contributed to senescence [55]–[57], raising the question if PR5 proteins are indeed pleiotropic. So far, induction of PR proteins has been considered as a terminal event in the signal transduction cascade. Our studies suggest that it may not be the case and that PR5 proteins may also indirectly contribute to other defense regulatory mechanisms such as phenylpropanoid and phytoalexin pathways.

## Materials and Methods

### Plant materials

European plum (*Prunus domestica* L.) fruits used in this study were collected from mature trees [25 yrs old] maintained at the Experimental Station, University of Guelph, Vineland, Ontario, Canada. The *Arabidopsis thaliana* wild type (WT) and transgenic plants used throughout this work were on the Columbia (Col-0) ecotype. Plants were grown in conviron chambers under a 16-h photoperiod at 25°C.

### Fungal inoculation

For *in vitro* fungal inoculation studies, fruits were harvested at commercial maturity and inoculated with 20 µl of *M. fructicola* conidial suspension (1×10^3^ conidia ml^−1^) or 20 µl of water (mock inoculation). After treatment, the fruits were incubated at room temperature in clear tote boxes with lids. Sterilized paper towels drenched with sterile water were placed inside the totes to increase humidity and favor fungal infection. Skin and flesh from the infected area were carefully excised and frozen immediately in liquid nitrogen. All such frozen material was stored at −80°C until further analysis.

*Alternaria brassicicola* infections were carried out on fully expanded leaves obtained from four-week-old ‘Columbia’ (wild type) and transgenic *Arabidopsis* plants harbouring the plum *PdPR5-1* gene. A virulent strain of *A. brassicicola* strain MUCL 20297 (kindly provided by Dr. M.R. McDonald, University of Guelph, Canada) was used in this study. The fungus was grown on potato dextrose agar (Sigma, Canada) medium at 22°C with 125 µM m^2^ s^−1^ cool-white fluorescent illumination on a 12-h-light/12-h-dark cycle for 9 d. Subsequently, the spores were collected from the plate. Concentration of spores was adjusted to 5×10^5^ spores mL^−1^. The sixth through 11th rosette leaves were detached from the WT and the transgenic *Arabidopsis* plants and inoculated by placing droplets of 5 µL of suspension onto leaf surface and water was used for mock treatment. Inoculated leaves were kept at 100% RH at room temperature.

### RNA extraction and cDNA synthesis

*P. domestica* fruit tissues were ground in liquid nitrogen using 6750 SPEX Sampleprep Freezer/Mill (SPEX certiprep, Metuchen, NJ, USA). Total RNA was extracted from two grams of fruit tissues, as described by [58]. Total RNA was isolated from *Arabidopsis* plants as described by [59]. DNA was removed from the samples using the RNase free DNaseI treatment according to the manufacturer's instructions (Promega, Madison, WI, USA) followed by a cleanup with RNeasy mini kit (Qiagen, Mississauga, ON, Canada). Two micrograms of DNase-treated RNA was used to synthesize the first strand cDNA using a random oligo *dt* primer and M-MuLV reverse transcriptase (New England Biolabs, Vancouver, BC, Canada) according to manufacturer's instructions.

### Gene cloning and constructs preparation

First strand reaction product from the cDNA obtained above was used to clone a full length PR5 gene from plums. Based on the homology between the previously cloned PR5 genes from other *Prunus* species, we designed the primers (5′-ATGGGTGTCTTCACATATGAGAG-3′) and (5′-TTAGTTGTAGGCATCGGGGTG-3′) to amplify target gene. The obtained PCR product was cloned in pGEMT easy vector (Promega Madison, WI, USA) and used for sequencing [Genbank: HM853674]. Plum genomic DNA was isolated from immature leaves using the CTAB method and used to clone the promoter region of the *PdPR5-1* by using the Universal Genome Walker Kit (Clontech, Palo Alto, CA. USA) according to the manufacturer's guidelines. All the sequencing in this study was carried out by Macrogen, Seoul, S. Korea. To prepare the *PdPR5-1* construct for stable transformation, the full length coding region of *PdPR5-1* was amplified by polymerase chain reaction (PCR) to introduce a BamHI site at both the 5′ and 3′ ends using I-Proof Taq Polymerase (BioRad, Mississauga, ON, Canada). The PCR products were digested with BamHI and ligated into BamHI site of the binary vector under control of the Cauliflower mosaic virus (CaMV) 35S promoter to yield plasmid construct 35S::*PdPR5-1*.

### *Arabidopsis* transformation

The plasmid 35S::*PdPR5-1* was introduced into *Agrobacterium tumefaciens* strain C58C1 and used to transform *Arabidopsis* using floral dip method [60]. Putative transformants were selected by plating the collected seeds on Murashige & Skoog medium containing 50 mg/L kanamycin. The kanamycin-resistant seedlings, identified by their dark green cotyledonary leaves and uninhibited growth thereafter, were transferred to soil. Presence of transgene (PR5) was confirmed in these plants by PCR.

### Quantitative RT-PCR

Real-Time PCRs were performed using the equivalent of 5 ng of total RNA in a 20 µl total reaction volume using 0.75 X of cyber green fluorescence. Specific primers and the accession number for the genes that were used to perform the reactions are provided in Table 3. All RT-PCR experiments were run in triplicate. Relative fold differences were calculated based on the comparative Ct method using actin or tublin of respective species as an internal standard and the 2^ΔΔCt^ formula.

**Table 3:** Primer sequences used in performing the real time PCR.

Primer Name	Sequences	Accession number
AtTUB4F	5′ TGCTGTCTTCGTTTCCCTGG 3′	M21415
AtTUB4R	5′ GAGGGTGCCATTGACAACATC 3′	
psActin-F	5′ CTGGACCTTGCTGGTCGT 3′	EF585293
psActin-R	5′ ATTTCCCGCTCAGCAGTG 3′	
at* CYP79B2F*	5′ ATGCTCGCGAGACTTCTTCAAGGT 3′	AT4G39950
at* CYP79B2*R	5′ AGATGCTCCGGCAATCTAAGGTCA 3′	
at *CYP79B3*F	5′ GTGATCACACCGTTTGGCGAACAA 3′	AT2G22330
at *CYP79B3*R	5′ TGTAGAGCCAAGCGGTCAAGTGAT 3′	
AtPADF	5′ TGACCGAGCTGATCAGAAACCCAA 3′	AT3G26830
AtPADR	5′ TCTTGGGAGCAAGAGTGGAGTTGT 3′	
atPAL2F	5′ ATCTGGAATGGCGTCGATGGTTCT 3′	L33678
atPAL2R	5′ ACCTCCGCGAAGATCGCTGATAAA 3′	
PdPR5-1F	5′-AGTCCTCAGCCTCAGCTTAACCAT-3′	HM853674
PdPR5-1R	5′-TTGGGATGCTAACTCGAACCCTGT-3′	

### Camalexin estimation

Camalexin from *Arabidopsis* leaf tissue was analysed based on the procedure developed by [49], using purified camalexin standard (kindly provided by Dr. Jane Glazebrook, Department of Plant Biology, University of Minnesota, USA). 200 mg of the plant tissue was extracted in 200 µl of 80% methanol at 60°C. 1 µl of the sample was further diluted 1000 times before direct injection MS analysis. MS analyses were carried out on an ion trap mass spectrometer (ABI Q-Trap 4000 system, A) equipped with a linear ion trap. The solution used for washing the autosampler syringe and injection needle was acetonitrile. Mobile phase A was composed of water acidified with acetic acid (pH 3) and mobile phase B was composed of 100% acetonitrile. The ion trap mass spectrometer was operated in the positive ion mode with the ion source voltage set to 5.5 kV, capillary temperature 250°C, sheath gas 40 (arbitrary units) and auxiliary gas 40 (arbitrary units). Mass spectra were acquired over the scan range m/z 100 to 1000 and collected with a step size of 0.1 amu. Declustering potential was 41 and curtain gas of 10 (arbitrary units). Collision-induced dissociation (CID) experiments used helium as the collision gas and collision energy was 50. Multiple reaction monitoring was performed for products of 201.1 for quantification. The prominent peak with m/z 59 was used for relative quantification of camalexin. The data were normalized to percentage per maximum intensity. A standard graph was plotted for camalexin with concentrations ranging from 1 ng to 5 ng. The linear equation was then used to calculate the absolute values for camalexin concentrations for the different samples.
